# The Effects of Osteopathic Manipulative Treatment (OMT) on Postoperative Length of Stay: A Meta-Analysis

**DOI:** 10.7759/cureus.59983

**Published:** 2024-05-09

**Authors:** Luke Henwood, Monique E Le Donne, Austin Vaughn, Shahir Kamil, Ava Harrington, Randy Scott

**Affiliations:** 1 Osteopathic Medicine, Lake Erie College of Osteopathic Medicine, Bradenton, USA; 2 Family Medicine, Lake Erie College of Osteopathic Medicine, Bradenton, USA

**Keywords:** holistic care of patients, post-sternotomy, arthroplasty, coronary artery bypass graft (cabg) surgery, ambulation, osteopathic manipulation, post-operative pain management, post-operative ileus, post-operative management, length-of-stay

## Abstract

Osteopathic manipulative treatment (OMT) is a therapy used by osteopathic physicians in various medical settings. Postoperatively, OMT can be utilized to optimize the body's function and recovery. This meta-analysis examines the efficacy of OMT in reducing the length of postoperative hospital stays. Given the significant implications of prolonged hospitalization for both patients and healthcare resources, research strategies to safely shorten this period are crucial. This meta-analysis examined five select studies that measured the length of hospital stay in postoperative patients who received OMT compared with postoperative patients who did not. A random effects model was applied in our statistical analysis to account for heterogeneity due to variations in surgical procedures, hospitals, and patient populations. Individually, three studies reported statistically significant reductions in hospital stay for OMT patients, while two did not. This meta-analysis, comprising five studies and 519 patients, found a mean difference of -2.37 days in favor of OMT; however, this finding did not reach a statistical significance (P = 0.06). The substantial heterogeneity observed (heterogeneity tau^2^ = 6.75, chi^2^ = 34.6, df = 4, P < 0.00001, I^2^ = 88%) suggests that clinical dissimilarities among the five studies may have resulted in our inconclusive findings. While OMT shows promise in postoperative care, further research with standardized protocols and more homogenous patient populations is needed to assess its true impact.

## Introduction and background

Osteopathic manipulative treatment (OMT) utilizes various techniques to align the human body and optimize its function and recovery [1]. In hospital settings, OMT has the potential to reduce the length of stay (LOS) for postoperative patients [2]. This meta-analysis aims to examine the efficacy of OMT in decreasing postoperative LOS, focusing on the core fundamental principles of osteopathy: the body as a unit, the body is capable of self-healing, and the reciprocal relationship between body structure and function [1].

The duration of hospital stay following surgery holds significant implications for both patients and hospital resources [3]. Consequently, surgeons and medical professionals strive to safely discharge patients home in an efficient manner. However, complications such as postoperative ileus pose a challenge for timely surgical patient discharge. Postoperative ileus refers to the obstruction of stool passage after surgery, which can be attributed to the disruptive effects of general anesthesia on the autonomic nerve system [4]. This presents an opportunity to explore the benefits of incorporating OMT in postoperative care. Osteopathic treatment of postoperative ileus has been well-studied and is just one example of postoperative OMT utility. Other postoperative uses of OMT found in the literature include but are not limited to increased mobility and ambulation [5], improved lung function, and reduced pain intensity reported by patients [6]. This meta-analysis aims to investigate the impact of OMT on LOS among postoperative patients who underwent either abdominal or cardiothoracic surgery.

## Review

Methods

The Search for Articles

A comprehensive search of electronic databases, including Google Scholar, PubMed, Science Direct, and Elsevier, was performed. These databases were selected to ensure comprehensive coverage of relevant literature. Online journals searched included but were not limited to the International Journal of Osteopathic Medicine, The American Academy of Osteopathy, and the Journal of the American Osteopathic Association. A combination of relevant keywords and terms was used to search for relevant articles. The search terms included “length of stay”, “early”, “delayed”, “abdominal surgery”, “thoracic surgery”, “OMT”, and “postoperative OMT”. Our search was limited to studies published in English in a full-length format. Only studies that recorded the mean length of hospital stay were included. Many studies were excluded because they examined other effects of postoperative OMT such as postoperative ileus or analgesic use, but did not report LOS. One study was excluded because LOS was only reported as a median value, not as a mean, and no raw data were provided to adjust for comparison with the other studies. Studies were either randomized controlled trials or retrospective cohort studies. Ideally, only randomized controlled trials would have been used for meta-analysis, but this was limited by the number of available publications.

**Figure 1:**
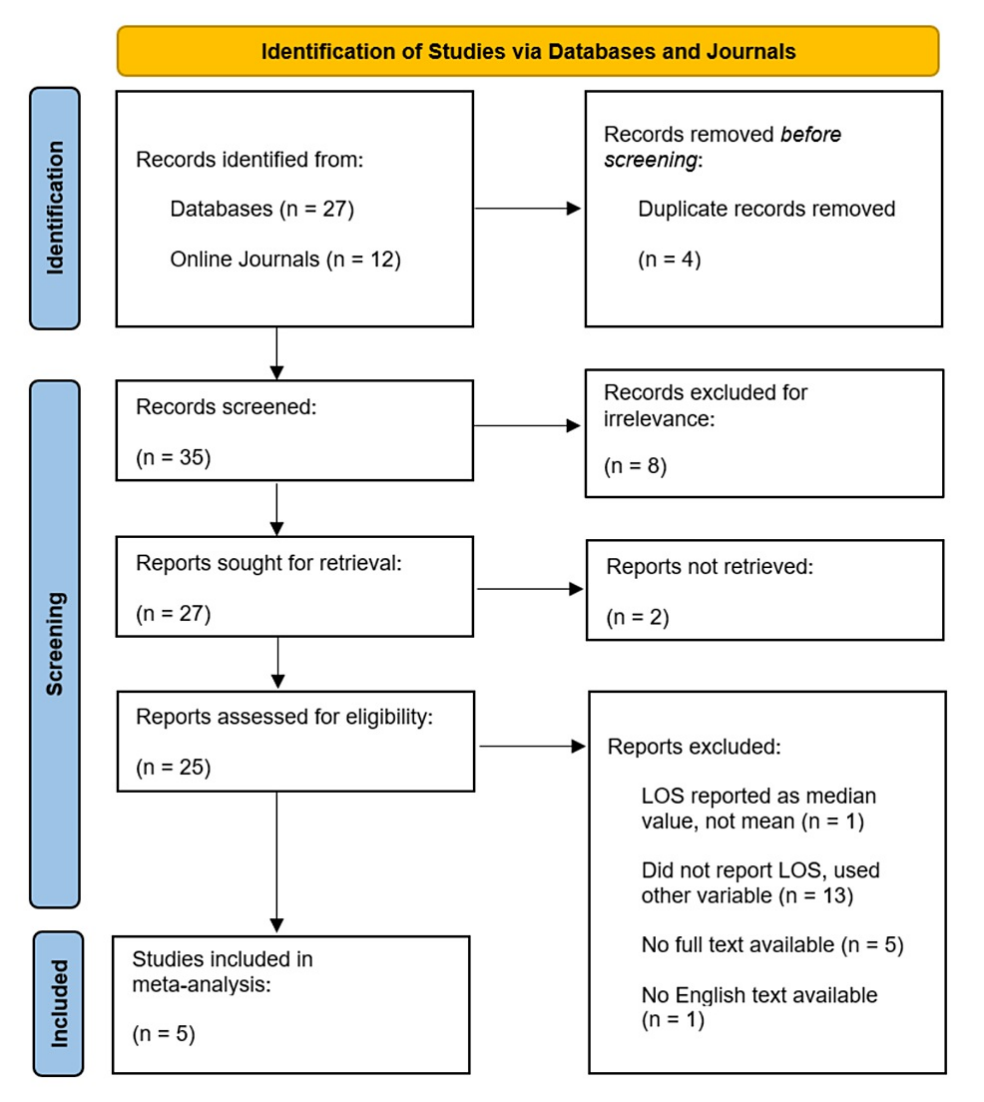
Flow diagram of the inclusion criteria of studies eligible for meta-analysis.

We collectively compiled and screened 39 articles from databases and online journals based on the aforementioned inclusion criteria. Any discrepancies were thereafter discussed, which then narrowed our list down to five (see Figure 1). The final list of articles compared LOS after abdominal and thoracic surgeries for patients receiving standard care versus standard care plus OMT (Table 1). Data extraction was performed, and the following information was collected from each study: author and publication year, number of patients, study method, type of surgery, effects measured, anatomical regions for OMT, OMT techniques, and relevant statistical data (Table 1). It should be noted that Wieting et al. included a placebo OMT group of 18 patients alongside the OMT and control groups [7]. No other study reviewed included a placebo OMT group. For streamlining purposes, data collected for the OMT placebo group in Wieting et al. were not used in this meta-analysis and are not included in our total patient number (n = 519).

**Table 1:** Characteristics of the studies included in the meta-analysis. ^*^n = 53 patients in Wieting et al. represent their total number of patients [7]. We excluded 18 of those 53 patients due to receiving placebo treatment. After the exclusion of the placebo group, the total number of patients from Wieting et al. [7] included in our analysis is n = 35. CABG = coronary artery bypass graft; BM = bowel movement; RCT = randomized controlled trial; LOS = length of stay; ME = muscle energy; MFR = myofascial release; BLT = balanced ligamentous tension

Study	Total Participants (n)	Method	Type of Surgery	Effects Measured	Regions Treated	OMT Technique
Racca et al., 2017 [6]	80	RCT	Sternotomy, CABG, valve replacement, aortic surgery	LOS, pain, respiratory functional capacity, analgesic intake, anxiety, depression,	Diaphragm, sternum, thoracic inlet	MFR, soft tissue
Wieting et al., 2013 [7]	53^*^	RCT	CABG	LOS functional status, time to BM	Ribs, thoracic spine, cervical spine, thoracic inlet	MFR, rib raising, soft tissue, suboccipital release
Fleming et al., 2015 [8]	38	Retrospective Cohort	Thoracotomy with lung resection	LOS, number of patients diagnosed with post-op ileus on discharge, number of patients receiving each type of osteopathic manipulative treatment	Cervical spine, thoracic spine, ribs, cranium, abdomen, lumbar spine, sacrum, upper extremity, lower extremity, pelvis	MFR, BLT, ME, rib raising
Crow et al., 2009 [9]	311	Retrospective Cohort	Abdominal	LOS	Cranium, cervical spine, thoracic spine, ribs, diaphragm, mesentery, celiac ganglion, superior mesenteric ganglion, inferior mesenteric ganglion, lumbar spine, sacrum, pelvis, and thoracic duct	Not listed. Regions treated were determined by somatic dysfunctions found on physical exam
Baltazar et al., 2013 [10]	55	Retrospective Cohort	Gastrointestinal	LOS, time to flatus, time to BM, time to clear liquid diet	Cranium, ribs, thoracic, and cervical spine	Variety including cranial, ME, and MFR, but not directly reported

Statistics

LOS was reported in each of the five chosen studies, measured in days. Since LOS is a continuous variable and was measured with consistent methods across all studies, the mean difference was used as a measure of effect. Review Manager 5.4.1 software (Cochrane Collaboration, Oxford, UK) was used to calculate 95% confidence intervals and apply weight to each study according to a random effects model. A random effects model was used to assume heterogeneity between studies given that each study performed different surgeries on their patient groups in different hospitals with varying patient populations. Heterogeneity was assessed by calculating the I^2^ statistic. A Z-test was performed to test for the overall effect.

Individually, three of the five studies (Racca et al. [6], Crow et al. [9], and Baltazar et al. [10]) showed a statistically significant reduction in postoperative LOS in the OMT treatment group. Fleming et al. [8] did not show a statistically significant reduction in LOS. This is possible because the patients in the OMT group had a greater “severity of illness” and 67% of the OMT group had two thoracic surgeries instead of one as compared with the control group [8]. Wieting et al. [7] found that OMT patients were “discharged 0.55 days earlier than those in the control group,” but this was not statistically significant (P = 0.49) [7]. Wieting et al. [7] did not provide any reasoning for why their findings did not reach statistical significance.

Results

**Figure 2:**
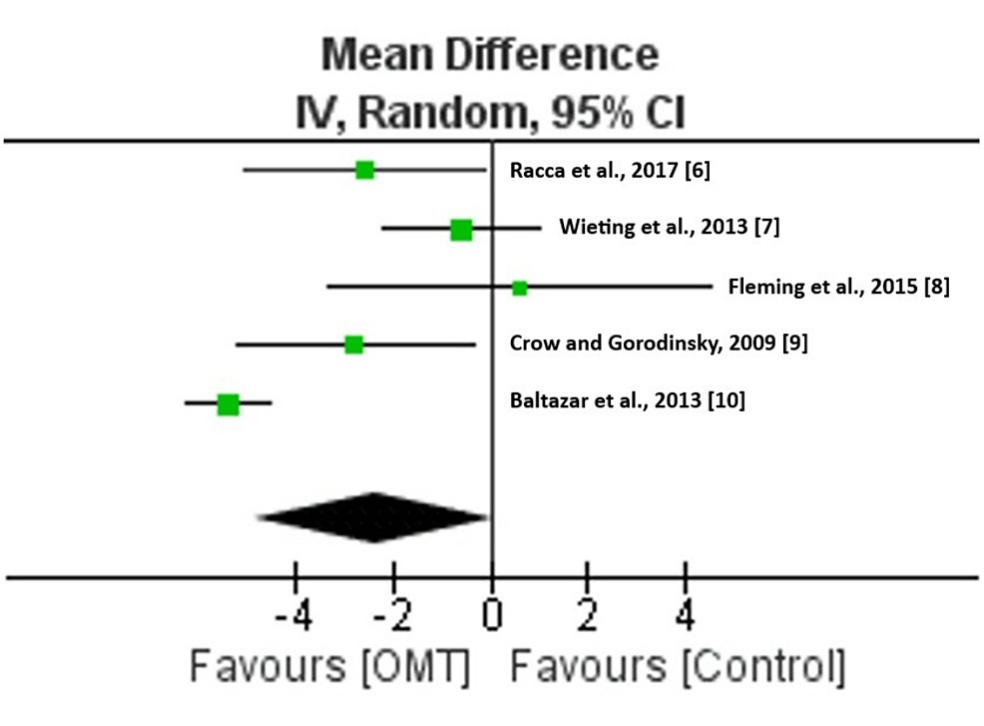
Length of hospital stay following postoperative OMT versus control forest plot. The forest plot was created using the data shown in Table 2 with Review Manager 5.4.1 software. The summary effect diamond crosses the line of no effect.

Our meta-analysis of 519 patients from five studies found that OMT did not statistically reduce postoperative LOS (MD: -2.37; 95% CI: -4.88, 0.13; Z = 1.86; P = 0.06). The I^2^ value of 88% and P < 0.00001 reject the hypothesis of no heterogeneity and actually suggest “substantial heterogeneity” [11]. Heterogeneity describes the variations in results between studies not due to chance alone, such as differences in subjects, treatment, and overall methods. These results may indicate that the studies chosen in this study are not similar enough clinically to be used for meta-analysis. Clinical dissimilarities leading to our inconclusive results may include different surgeries performed, different surgical techniques, varied patient health status, different hospital locations, etc. Table 2 displays relevant data collected among the five studies as well as the weight applied to each study. Figure 2 is a forest plot made from the data listed in Table 2 using Review Manager 5.4.1 software. The summary effect diamond in Figure 2 crosses the line of no effect, indicating that there is no statistical significance in favor of OMT reducing postoperative LOS.

**Table 2:** Meta-analysis of the length of hospital stay following postoperative OMT. Heterogeneity tau^2^ = 6.75. chi^2^ = 34.6. df = 4 (P < 0.00001). I^2^ = 88% Test for overall effect Z = 1.86 (P = 0.06) CI = confidence interval; SD = standard deviation "-" = no data calculated as total using 95% confidence interval was only calculated for the total number of patients

Study, Year	Mean [Days] (OMT)	SD [Days] (OMT)	Total Patients (OMT)	Mean [Days] (Control)	SD [Days] (Control)	Total Patients (Control)	Weight (%)	IV, Random, CI 95%
Racca et al., 2017 [6]	19.1	4.8	40	21.7	6.3	40	19.6	-2.60 [-5.05, -0.15]
Wieting et al., 2013 [7]	6.1	1.7	17	6.7	3	18	22.0	-0.60 [-2.14, 0.94]
Fleming et al., 2015 [8]	11	6.8	23	10.4	5.5	15	15.2	0.60 [-3.33, 4.53]
Crow et al., 2009 [9]	11.8	10.71	172	14.6	11.13	139	19.7	-2.80 [-5.25,-0.35]
Baltazar et al., 2013 [10]	6.1	1.7	17	11.5	1	38	23.5	-5.40 [-6.27, -4.53]
Total (95% CI)	-	-	269	-	-	250	100	-2.37 [-4.88, 0.13]

Discussion

When holistically treating a surgical patient, it is important to consider both pre- and postoperative management. Hospital LOS can alter a patient's disease process and post-surgical healing, making it a valuable objective measurement for analyzing postoperative care. Increased LOS is known to be associated with negative outcomes such as infection, increased mortality risk, increased fall risk, slower recovery, and increased healthcare cost, thereby affecting all aspects of medicine from patient care to healthcare provision [12]. The use of OMT in postoperative patients has been shown in the literature to be a valuable, inexpensive tool when attempting to reduce the length of hospitalization, reduce pain, and increase positive outcomes [8].

OMT can be performed in a variety of ways, and many osteopathic physicians add their own style to traditionally learned methods, making it challenging to streamline OMT for research purposes. OMT consists of both active and passive techniques, in which the active form involves the patient’s assistance and the passive form is purely performed by the physician. In the immediate postoperative setting, passive treatments may be easier to conduct than active treatments due to the patient's pain level and the lasting effects of anesthesia. OMT can also be categorized as direct or indirect, meaning the technique is guided directly towards the area of discomfort (functional barrier) or indirectly into the ease of the dysfunction [2,5]. Indeed, it is important to consider the limitations and contraindications of OMT in the postoperative period. Concerns about postoperative OMT often include surgical incisions, wounds, non-weight bearing limitations, and casted extremities. It is not surprising that the majority of OMT used in the five studies (see Table 1) included in this meta-analysis consisted of passive techniques such as myofascial release, cranial manipulation, and rib raising. Given the variety of techniques available, OMT can be a safe, gentle, and effective way to reach postoperative recovery.

Post-gastrointestinal surgery OMT was examined in several studies in the literature [9,10,13-15]. Some of the OMT used in those studies included the CV-4 technique, point of balance fascial tension of the colon, neural inhibition for the intestines, high-velocity low amplitude (HVLA), myofascial release, articulatory techniques, and muscle energy [9,10,13-15]. A common theme among the studies used in this meta-analysis and other studies in the literature is that the techniques chosen were mostly based on physician preference and patient tolerance [6-10,13-15]. This demonstrates that OMT can be adapted for individual patient needs and safety postoperatively. Pratt-Harrington et al. found that the use of OMT in post-abdominal surgery patients promoted significant improvement in patients’ incentive spirometry measurements, suggesting that OMT could reduce the risk of atelectasis and pneumonia and improve overall breathing for earlier hospital discharge [14]. This study exemplifies the effectiveness of postoperative OMT in an anatomical region that was not operated on (i.e., abdominal surgery and breathing treatment). Similarly, a case report by Ridgeway et al. found that OMT treatment to the cranial, costal, thoracic, sacral, and abdominal regions decreased pain and increased range of motion in a postoperative sigmoid colon resection patient [15]. Treating the body as a whole in these aforementioned studies using OMT improved patient function and contributed to reduced hospitalization time [13-15].

In addition to gastrointestinal surgery, OMT has also been shown to improve outcomes and decrease LOS following cardiothoracic surgeries. Crow et al. studied 80 post-sternotomy patients and found that OMT used in conjunction with standard treatment significantly lowered pain and decreased LOS when compared to those who did not receive OMT [9]. Additionally, Wieting et al. found that those who received OMT treatment following coronary artery bypass graft (CABG) surgery were discharged 0.55 days earlier than control groups who did not receive OMT and 0.16 days earlier than placebo groups [7]. Each of these studies suggests that OMT can potentially decrease the LOS in postoperative cardiothoracic surgery patients.

OMT is also efficacious in improving postoperative outcomes among orthopedic surgery patients undergoing arthroplasty. Zhou et al. found that OMT increased the range of motion and decreased the need for analgesics following total knee arthroplasty [5]. OMT shortened LOS by allowing patients to better perform activities of daily living. When comparing the OMT group versus the non-OMT group, the OMT group had more efficient stair climbing mobility and ambulation, both of which are related to patient satisfaction and recovery [5]. From this, OMT-treated patients were able to move earlier and thus be discharged sooner. As with the other aforementioned studies, Zhou et al. used different OMT modalities according to individual patient needs, which limits the ability to assess which OMT techniques were most efficacious in decreasing LOS. However, there seems to be a trend in the literature demonstrating that OMT can reduce LOS, no matter the modality used or the type of surgery performed. 

As previously discussed, safely reducing the LOS benefits the patient, provider, and overall hospital system. Not only does the patient's health benefit but their financial expenses are substantially decreased. For example, Mao et al. followed 88 patients with postoperative ileus and found that their cost was 71% higher than those without postoperative ileus due to increased LOS [4]. OMT effectively treated patients with postoperative ileus in two of the studies included in this meta-analysis [9,10]. Crow et al. found that treatment of postoperative ileus with OMT lessened the LOS by 2.8 days versus those not treated with OMT [9]. Shortened LOS in OMT-treated postoperative ileus patients was also true for Baltazar et al. [10]. All the aforementioned studies exemplify how OMT can be an effective tool in postoperative recovery. However, as shown in the literature and in the results of this meta-analysis, more research needs to be done using streamlined OMT treatments for specific surgeries. It is important to note this article was written by authors in the field of osteopathic medicine, and as such, there is potential for bias. Every attempt to minimize this bias was considered, and this article is meant to expand research in the field of OMT and its effect on LOS. Our meta-analysis has several limitations, including unequal OMT treatments, unequal surgical procedures, and of course varied hospital settings. Due to these limitations, heterogeneity was assumed when performing statistical analysis and was found to be substantial (I^2 ^= 88%). This study was also limited by the small number of publications in the literature measuring postoperative LOS as an effect of OMT. Due to these limitations, further research on postoperative OMT and LOS is needed to conduct a meta-analysis on a larger scale.

## Conclusions

This meta-analysis examined the impact of OMT on postoperative LOS across five selected studies. The mean difference of postoperative LOS in groups receiving OMT versus those who did not was calculated using a random effects model to account for heterogeneity. Three of the five individual studies reported a statistically significant reduction in postoperative LOS for OMT-treated patients, while two studies did not demonstrate a significant effect.

Our meta-analysis of five studies, including 519 patients, yielded a mean difference of -2.37 days in favor of OMT, but this did not reach statistical significance (P = 0.06). The substantial heterogeneity observed, with an I^2^ value of 88%, suggests clinical dissimilarities in patient populations, surgical treatment, and methodology between the five studies. These results highlight the importance of clinical context and methodological nuances when assessing the potential benefits of OMT on postoperative LOS. Further research with standardized OMT protocols and more homogenous patient populations is warranted to evaluate the true effect of OMT in postoperative care.

## References

[1] Roberts A, Harris K, Outen B (2022). Osteopathic manipulative medicine: a brief review of the hands-on treatment approaches and their therapeutic uses. Medicines (Basel).

[2] Heineman K (2022). An osteopathic manipulative treatment (OMT) evaluation and treatment protocol to improve gastrointestinal function. AAO Journal.

[3] Rojas-García A, Turner S, Pizzo E, Hudson E, Thomas J, Raine R (2018). Impact and experiences of delayed discharge: a mixed-studies systematic review. Health Expect.

[4] Mao H, Milne TG, O'Grady G, Vather R, Edlin R, Bissett I (2019). Prolonged postoperative ileus significantly increases the cost of inpatient stay for patients undergoing elective colorectal surgery: results of a multivariate analysis of prospective data at a single institution. Dis Colon Rectum.

[5] Zhou Y, Chin J, Evangelista A, Podger B, Wan PJ, Lomiguen CM (2022). Inhibiting the musculoskeletal pathological processes in post-knee replacement surgery with osteopathic manipulative treatment: a systematic review. Cureus.

[6] Racca V, Bordoni B, Castiglioni P, Modica M, Ferratini M (2017). Osteopathic manipulative treatment improves heart surgery outcomes: a randomized controlled trial. Ann Thorac Surg.

[7] Wieting JM, Beal C, Roth GL, Gorbis S, Dillard L, Gilliland D, Rowan J (2013). The effect of osteopathic manipulative treatment on postoperative medical and functional recovery of coronary artery bypass graft patients. J Am Osteopath Assoc.

[8] Fleming RK, Snider KT, Blanke KJ, Johnson JC (2015). The effect of osteopathic manipulative treatment on length of stay in posterolateral postthoracotomy patients: a retrospective case note study. IJOM.

[9] Crow WT, Gorodinsky L (2009). Does osteopathic manipulative treatment (OMT) improve outcomes in patients who develop postoperative ileus: a retrospective chart review. Int J Osteopath Med.

[10] Baltazar GA, Betler MP, Akella K, Khatri R, Asaro R, Chendrasekhar A (2013). Effect of osteopathic manipulative treatment on incidence of postoperative ileus and hospital length of stay in general surgical patients. J Am Osteopath Assoc.

[11] Dettori JR, Norvell DC, Chapman JR (2022). Fixed-effect vs random-effects models for meta-analysis: 3 points to consider. Global Spine J.

[12] Tipton K, Leas BF, Mull NK (2021). Interventions to decrease hospital length of stay. Agency for Healthcare Research and Quality (US).

[13] Mills M, Sevensma K, Serrano J (2020). Osteopathic manipulative treatment for a recognizable pattern of somatic dysfunction following laparoscopic cholecystectomy. J Am Osteopath Assoc.

[14] Pratt-Harrington D, Neptune-Ceran R (1995). The effect of osteopathic manipulative treatment in the post abdominal surgical patient. AAO Journal.

[15] Ridgeway V, Berkowitz M (2010). Somatic dysfunction following sigmoid colon resection for diverticulitis: a case report. AAO Journal.

